# Identification of Central Regulators of Calcium Signaling and ECM–Receptor Interaction Genetically Associated With the Progression and Recurrence of Atrial Fibrillation

**DOI:** 10.3389/fgene.2018.00162

**Published:** 2018-05-16

**Authors:** Petra Büttner, Laura Ueberham, M. B. Shoemaker, Dan M. Roden, Borislav Dinov, Gerhard Hindricks, Andreas Bollmann, Daniela Husser

**Affiliations:** ^1^Department of Electrophysiology, Heart Center Leipzig, Leipzig University, Leipzig, Germany; ^2^Department of Medicine, Vanderbilt University Medical Center, Nashville, TN, United States

**Keywords:** protein–protein interactions, genetic variants, atrial fibrillation, atrial fibrillation recurrence, atrial fibrillation progression, calcium signaling, ECM–receptor interaction

## Abstract

Atrial fibrillation (AF) is a multifactorial disease with a strong genetic background. It is assumed that common and rare genetic variants contribute to the progression and recurrence of AF. The pathophysiological impact of those variants, especially when they are synonymous or non-coding, is often elusive and translation into functional experiments is difficult. In this study, we propose a method to go straight from genetic variants to defined gene targets. We focused on 55 genes from calcium signaling and 26 genes from extra cellular matrix ECM–receptor interaction that we found to be associated with the progression and recurrence of AF. These genes were mapped on protein–protein interaction data from three different databases. Based on the concept that central regulators are highly connected with their neighbors, we identified central hub proteins according to random walk analysis derived scores representing interaction grade. Our approach resulted in the identification of *EGFR*, *RYR2*, and *PRKCA* (calcium signaling) and *FN1* and *LAMA1* (ECM–receptor interaction) which represent promising targets for further functional characterization or pharmaceutical intervention.

## Introduction

Common genetic variants contribute to the progression and recurrence of atrial fibrillation (AF; Olesen et al., 2014). We recently used genome-wide association study data from 660 AF patients to detect common variants that associate with left atrial diameter, AF type (paroxysmal vs. persistent AF), and AF recurrence (Husser et al., 2016a,b). Based on the hypothesis that all genetic variants irrespective of their significance level contribute to genetic background, we included all variants with *p* < 0.05 in our analysis irrespective if they were coding, non-coding, synonymous, or non-synonymous. Using a stepwise filtering procedure, we shifted the conventional SNP-based analysis toward a gene-based analysis (Li et al., 2011; Chanda et al., 2013) and finally tested the genes for non-random enrichment in physiological pathways. This approach revealed an association of calcium signaling (55 genes) and ECM–receptor interaction (26 genes) with left atrial diameter and AF type, respectively, and also with AF recurrence (Husser et al., 2016a; see Supplementary Table S1). Abnormal intracellular Ca(2+) load, distribution, and handling are involved in AF initiation, maintenance, and progression (Nattel and Dobrev, 2012) as well as heterogeneous conduction slowing and reentry (Yue et al., 2011). Ca(2+) influx into atrial fibroblasts triggers differentiation into ECM-remodeling myofibroblasts which in turn trigger atrial fibrosis, the basis of electroanatomical remodeling, and AF maintenance and progression (Yue et al., 2011). Cardiac fibrosis is furthermore characterized by altered ECM–receptor interactions of cell–cell contacts and cell–matrix adhesions involving integrins, fibronectin, collagen, and laminin (Giancotti and Ruoslahti, 1999; Schroer and Merryman, 2015).

Central pathway regulators represent promising targets for replication and explorative studies. *In silico* protein-network analysis can be used for the identification of these regulators. Protein–protein interaction (PPI) data from functional and bioinformatics experiments are available in PPI databases (Calderone et al., 2013; Alonso-Lopez et al., 2016; Fabregat et al., 2016) whereas their composition, complexity, and reliability differ. The mapping of candidate genes to PPI networks can be done using the freely accessible software tool Cytoscape (Shannon et al., 2003) and the application iPINBA (Wang et al., 2015).

Summarizing, we applied an approach putting together PPI data and the recently identified candidate genes from GWAS analysis to identify central regulators of calcium signaling and ECM–receptor interaction associated with AF progression and recurrence.

## Materials and Methods

This study was based on recently published findings (Husser et al., 2016a). Patient characteristics, clinical parameters, genome-wide association analysis, gene-based association testing, pathway analysis, and gene lists can be found in Supplementary Methods and Supplementary Table S1. The study protocol was approved by the local Ethics Committee. All patients signed written informed consent for study participation.

### PPI Network Analysis

Cytoscape v3.4.0 (Shannon et al., 2003) was used for PPI network construction. UniProt identifiers for all candidate genes were retrieved from UniProt ID mapping service^1^ (Supplementary Table S1). PPI data were imported via PSICQUIC client (Aranda et al., 2011) or manually from original repositories. The selection of a specific database introduces a bias as the PPI evidence criteria applied by the database curators differ. Therefore, we included three manually curated databases in our analysis to minimize bias.

We used data from Agile Protein Interactomes Data Server (APID; Alonso-Lopez et al., 2016), mentha PPI database (mentha; Calderone et al., 2013), and Reactome knowledgebase (Fabregat et al., 2016). Furthermore, APID, Reactome and mentha access data from other important PPI databases, namely, Molecular INTeraction database (MINT; Licata et al., 2012), IntAct Molecular Interaction Database (IntAct; Orchard et al., 2014), Database of Interacting Proteins (DIP; Salwinski et al., 2004), extracellular matrix interaction database (MatrixDB; Launay et al., 2015), BioPlex (Huttlin et al., 2015), and BioGRID (Chatr-Aryamontri et al., 2017). At the time of analysis (February 2017), mentha interactome comprised 259,599 interactions of 18,245 proteins, APID database comprised 349,144 interactions for 29,701 proteins, and Reactome included 221,866 interactions of 8631 proteins.

Cytoscape app iPINBPA (Wang et al., 2015) was used for further analysis. Candidate genes from calcium signaling and ECM–receptor interaction (Supplementary Table S1) were mapped on the three PPI networks to build sub-networks.

### Random Walk Analysis

To identify the central regulators in the sub-network, we applied random walk analysis. Random walk technique explores a network by simulating a walker who chooses randomly among available edges starting from one or many seed genes. Over time the walker will pass by all members in the network with different probabilities whereas highly connected nodes are more probably passed by several times (Can et al., 2005). All genes in sub-networks with more than two edges were assigned seed genes for random walk analysis. Random walk node weights (RWNW) calculated by iPINBPA were used to rank the candidate genes in descending order. Cytoscape network analyzer tool was used to determine the number of direct edges of every candidate gene. Sub-networks were visualized using Cytoscape.

We applied a two-step approach. First, we identified all candidate genes with more than two edges that were present among the top 10 RWNW rankings in every interactome (APID, mentha, and Reactome). This analysis was based on PPI data summarizing all reported interactions without evidence weighting including solely predicted interactions. Second, genes that passed step one were reanalyzed using more stringent PPI evidence filters according to APID level 2 PPI data (115,480 interactions of 16,016 proteins) summarizing only PPI validated in at least two independent experiments, e.g., co-expression, co-purification, co-crystallization, or yeast2hybrid (Alonso-Lopez et al., 2016).

## Results

**FIGURE 1:**
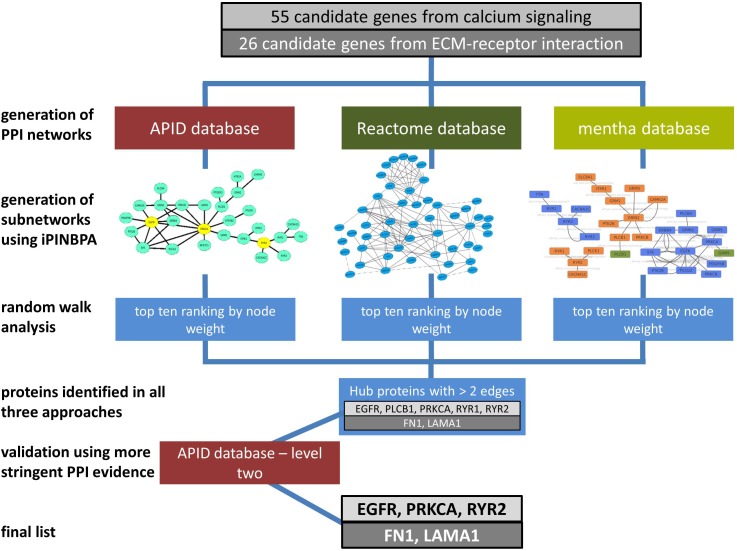
Flowchart of the stepwise approach that was used to identify central hub proteins out of 55 genes annotated to calcium signaling (light gray boxes) and 26 genes annotated to ECM–receptor interaction (dark gray boxes).

Genes from the pathways calcium signaling and ECM–receptor interaction, which we found associated with left atrial diameter increase, a switch from paroxysmal to persistent AF, and AF recurrence in a former study, were mapped on PPI data from three official databases, namely, APID, mentha, and Reactome. Sub-networks including all genes with at least two neighbors were created using iPINBPA. Nineteen (APID), 44 (Reactome), and 13 (mentha) genes from calcium signaling and eight (APID), 25 (Reactome), and six (mentha) genes from ECM–receptor interaction fulfilled these criteria. We next applied RWNW ranking to the sub-networks (**Table 1**). Finally, we identified those genes that ranked among the top 10 in all three sub-networks (flowchart depicted in **Figure 1**).

**Table 1:** Ranking of candidate proteins from **(A)** calcium signaling pathway and **(B)** ECM–receptor interaction pathway by random walk node weights (RWNW) using PPI data from three databases.

APID			Reactome			Mentha		
Gene	RWNW	Edges	Gene	RWNW	Edges	Gene	RWNW	Edges
**(A) Calcium signaling pathway**
***PRKCA***	0.062	10	*GNA14*	0.046	14	***EGFR***	0.079	13
***EGFR***	0.054	8	*CALML3*	0.030	12	***RYR2***	0.061	6
***RYR2***	0.049	4	***PRKCA***	0.029	10	***PRKCA***	0.057	5
*GNAQ*	0.045	3	***RYR2***	0.028	12	***PLCB1***	0.056	5
***RYR1***	0.045	3	*RYR3*	0.027	11	*GRIN1*	0.053	4
*GRIN1*	0.044	5	***RYR1***	0.027	11	***RYR1***	0.052	4
*ITPR1*	0.041	3	*PRKCB*	0.027	14	*PTGER3*	0.050	3
***PLCB1***	0.039	3	*GNAQ*	0.024	8	*SYK*	0.047	3
*SYK*	0.038	5	***EGFR***	0.024	8	*ERBB4*	0.044	2
*PRKCB*	0.035	4	***PLCB1***	0.023	11	*ATP2B4*	0.044	2
**(B) ECM–receptor interaction pathway**
***FN1***	0.137	8	***FN1***	0.0486	21	***FN1***	0.1227	5
*DAG1*	0.088	3	***LAMA1***	0.0470	19	*DAG1*	0.1077	4
***LAMA1***	0.081	3	*ITGB3*	0.0446	18	***LAMA1***	0.0885	3
*CD36*	0.069	3	*ITGA1*	0.0445	20	*SDC2*	0.0810	2
*SDC2*	0.069	2	*LAMC3*	0.0444	19	*ITGB3*	0.0810	2
*HSPG2*	0.060	2	*LAMA3*	0.0440	18	*HSPG2*	0.0781	3
*ITGB3*	0.059	2	*LAMA5*	0.0428	17	*LAMA3*	0.0660	1
*COL4A2*	0.059	2	*COL4A2*	0.0414	17	*CD36*	0.0660	1
*LAMA3*	0.057	1	*ITGA4*	0.0412	18	*ITGA1*	0.0627	1
*AGRN*	0.055	1	*ITGA9*	0.0412	18	*AGRN*	0.0619	1


*Candidate genes in bold were identified in every interactome.*

Epidermal growth factor receptor (*EGFR*), ryanodine receptor 2 (*RYR2*), phospholipase C, beta 1 (*PLCB1*), ryanodine receptor 1 (*RYR1*), and protein kinase C alpha (*PRKCA*) from calcium signaling and fibronectin (*FN1*) and Laminin subunit alpha-1 (*LAMA1*) from ECM–receptor interaction were identified to be hub proteins.

**FIGURE 2:**
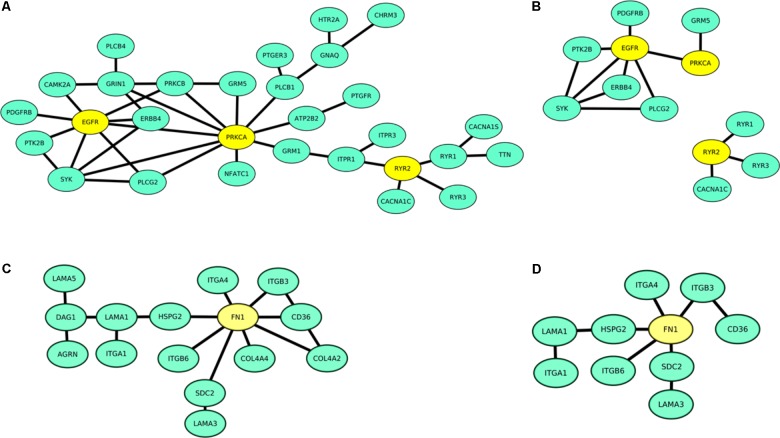
Protein interaction network of AF associated genes assigned to calcium signaling **(A,B)** and ECM–receptor interaction pathway **(C,D)** based on APID level 1 evidence level **(A,C)** and APID level 2 evidence level data which were validated at least twice **(B,D)**. Hub proteins are marked yellow.

We additionally repeated the analysis using more stringent APID level 2 assigned data, comprising only PPIs that were confirmed at least twice in independent experiments excluding predicted interactions. We confirmed all candidates except *PLCB1* and *RYR1* (**Figure 2**). The remaining candidates *EGFR*, *RYR2*, *PRKCA*, *FN1*, and *LAMA1* thus had at least two experimentally validated protein–protein physical interactions in the analyzed network.

## Discussion

Conventional GWAS identified genetic variants associated to AF by applying genome-wide significance level of 5 × 10^-8^. By analyzing their genomic loci, new candidate genes were identified, e.g., *PITX2*, *TBX5*, *ZFHX3*, and *KCNN3* (Fatkin et al., 2017). We applied a completely different approach using gene-based association testing and pathway enrichment and thus identified ECM–receptor interaction (26 genes) and calcium signaling (55 genes) to be most significantly associated with AF type, LAD increase, and AF recurrence (Husser et al., 2016a).

In this study, we aimed to develop a weighting method to identify promising candidate genes out of the unweighted gene lists. Our approach was based on the hypothesis that densely connected proteins in PPI networks, so-called hub proteins, are expected to be biologically essential proteins with the potential consequence that loss of these proteins is hardly tolerated by the organism what is referred to as “centrality-lethality rule” (Gursoy et al., 2008).

Our analysis was based on APID, Reactome, and mentha database as these reported at least 200 PPIs between the candidates of either pathway enabling a reasonable analysis. These PPI data comprise a heterogeneous collection of observations of different quality. We addressed this by utilizing and comparing three interactomes as we assumed that this approach decreases bias and increases reliability of results. For example, Reactome reported GNA14 and CALML3 to be among the mostly connected proteins in the analyzed network but this finding was not validated by APID and mentha data (see **Table 1**). We used random walk analysis to establish ranking scores indicating connectivity. Random walk analysis is a widely accepted method to explore networks and to identify highly connected nodes (Can et al., 2005; Huan et al., 2014). Additionally, we determined the number of direct edges. Ranking according to the number of edges would result in slightly different ranking of the genes, as in networks, central proteins are more likely passed by in random walk and are thus ranked higher than equally connected proteins at the rim (**Figure 2**). RWNW ranking therefore enables ranking of genes with equal numbers of direct edges. Our approach identified *EGFR*, *PRKCA*, and *RYR2* as central regulators of calcium signaling and *FN1* and *LAMA1* in ECM–receptor interaction. Involvement of the aforementioned genes in pathomechanism of arrhythmia was already partly examined.

EGF–receptor transactivation and dysregulation is involved in myocardial hypertrophy and contraction (Eguchi et al., 2013; Xu et al., 2014). In an animal model, EGF–receptor phosphorylation led to tyrosine phosphorylation of cardiac Na(+) and L-type Ca(2+) channels and thus modulated electrical excitability of the heart and ischemia/reperfusion associated cardiac arrhythmia (Feng et al., 2012).

*PRKCA* gene and protein expression was found upregulated in cardiac hypertrophy (Dorn and Force, 2005). Genetic variation in *PRKCA* was found to be associated with QRS duration (Sotoodehnia et al., 2010; Arking et al., 2014).

The main regulators of calcium release from the sarcoplasmic reticulum are ryanodine receptors whereas *RYR2* is the main cardiac isoform. Imbalances in RYR2 expression as well as genetic variants are associated with altered calcium handling and arrhythmia (Zhabyeyev et al., 2013; Di Pino et al., 2014; Li et al., 2014).

Fibronectin is a component of extra cellular matrix and *FN1* expression by fibroblasts was found to be increased in myocardial fibrotic remodeling and failing myocardium (Schaper et al., 2002; Fan et al., 2012). Levels of circulating fibronectin were found to be associated with atrial remodeling in AF (Canpolat et al., 2015).

Laminin is a major component of ECM, especially the basement membrane, and was found to be expressed significantly higher in the left atrium compared to left ventricle (Burstein et al., 2008). Laminin is involved in cardiac development and pathological remodeling (Schaper et al., 2002; Burstein et al., 2008).

### Limitations

The initial GWAS study design, resulting in the identification of AF associated calcium signaling and ECM–receptor interaction, was based on small sample size and a cross-sectional study design. We addressed this by analyzing well-defined phenotypes and applying a two-step approach (i.e., identification of pathways in two AF phenotypes and validation in a third phenotype).

We utilized and compared three interactomes. Far more PPI databases exist that are applicable for such an analysis and all PPI databases undergo constant editing suggesting that there is interesting developmental potential for the kind of analysis introduced by us.

Causative relationships of the identified regulators and AF progression and recurrence were not assessed and cannot be estimated from the underlying study design.

We are well aware that many more genes and pathways contribute to AF than we analyzed in our study (Fatkin et al., 2017). We started from pre-defined genes and pathways’ resulting from an innovative multi-step filter approach that was recently published by our group. Important candidates identified by other studies which are not part of calcium signaling and ECM–receptor interaction were beyond the focus of our study. *PITX2*, *ZFHX3*, and *KCNN3* were analyzed in a single gene approach as recently published by our group (Husser et al., 2017).

Finally, we want to point out that our gene-based analysis of GWAS data completely differs from conventional analysis with application of genome-wide significance cut off of at least 5E-8 for single SNPs. Gene-based analysis is a supplement but not a substitute for conventional analysis approaches.

## Conclusion

We identified *EGFR*, *PRKCA*, *RYR2* and *FN1*, *LAMA1* as central regulators of calcium signaling and ECM–receptor interaction associated with AF progression and recurrence. Further studies, especially functional analyses, should focus on the aforementioned central pathway regulators to elucidate the pathophysiological background of AF and their possible role as pharmacological targets.

## Author Contributions

DH, AB, and PB conceived and initiated the project, analyzed the data, and wrote the manuscript. LU and PB did the laboratory work. MS, DR, BD, and GH participated in data interpretation and critically revised the manuscript. All authors reviewed and contributed to the final manuscript, approved its publication, and are accountable for the content.

## Conflict of Interest Statement

The authors declare that the research was conducted in the absence of any commercial or financial relationships that could be construed as a potential conflict of interest.

## Footnotes

1 http://www.uniprot.org/uploadlists/
