# Supplementary material for: Identification of Central Regulators of Calcium Signaling and ECM–Receptor Interaction Genetically Associated With the Progression and Recurrence of Atrial Fibrillation
Source: Front Genet. 2018 May 16;9:162. doi: 10.3389/fgene.2018.00162 (PMC5964985; doi:10.3389/fgene.2018.00162)
Supplement: Supplementary file 1 [file Table_1.doc]

**Identification of central regulators of calcium signaling and ECM-receptor interaction genetically associated with the progression and recurrence of atrial fibrillation**

Petra Büttner*^1^, Laura Ueberham^1^, M. Benjamin Shoemaker^2^, Dan M. Roden^2^, Borislav Dinov^1^, Gerhard Hindricks^1^, Andreas Bollmann^1^, Daniela Husser^1^

^1^Department of Electrophysiology, Heart Center Leipzig, Leipzig University, Germany. ^2^Department of Medicine, Vanderbilt University, Nashville, TN, USA.

**Correspondence:**

Petra Büttner, PhD

E-Mail: [petra.buettner@medizin.uni-leipzig.de](mailto:petra.buettner@medizin.uni-leipzig.de)

**Supplementary Methods**

The following methods description is taken from our publication “Genomic contributors to rhythm outcome of atrial fibrillation catheter ablation - pathway enrichment analysis of GWAS data” published in PLOS ONE^1^.

**Patients**

Six hundred-and-sixty AF patients undergoing de-novo radiofrequency AF catheter ablation between 2008 and 2013 were enrolled in the Leipzig Heart Center AF ablation registry. Paroxysmal AF was defined as self-terminating episodes of AF within 7 days after onset documented by ECG or an ambulatory ECG monitor. Persistent AF was defined as an AF episode either lasting longer than 7 days or requiring drug or direct current cardioversion for termination.

In all patients, transthoracic and transesophageal echocardiography was performed prior to catheter ablation. Left atrial diameter and left ventricular ejection fraction were determined using standard measurements and a left atrial thrombus was excluded. All class I or III antiarrhythmic medications with the exception of amiodarone were discontinued at least 5 half-lives before the procedure.

The study protocol was approved by the local Ethics Committee. All patients signed written informed consent for study participation.

**AF catheter ablation and follow-up**

Patients were studied under deep propofol sedation with continuous invasive monitoring of arterial blood pressure and oxygen saturation. Non-fluoroscopic 3D catheter orientation, CT image integration, and tagging of the ablation sites were performed using Ensite NavX, Ensite Velocity (St. Jude Medical, St. Paul, MN, USA) or CARTO 3 (Biosense Webster, Diamond Bar, CA, USA). Trans-septal access and catheter navigation were performed with a steerable sheath (Agilis, St. Jude Medical, St. Paul, MN, USA). Patients presenting with AF at the beginning of the procedure were electrically cardioverted and ablation was performed during sinus rhythm (i.e. AF termination with ablation was not attempted). In all patients circumferential left atrial ablation lines were placed around the antrum of the ipsilateral pulmonary veins (irrigated tip catheter, pre-selected tip temperature of 48°C, and maximum power of 30 – 50 W). In patients with persistent AF, additional linear lesions were added at the left atrial roof, the basal posterior wall and the left atrial isthmus.

After circumferential line placement, voltage and pace mapping along the ablation line were used to identify and close gaps. The isolation of all pulmonary veins with bidirectional block was verified with a multipolar circular mapping catheter and was defined as the procedural endpoint.

After ablation, class I and III antiarrhythmic drugs were not reinitiated. Oral anticoagulation was prescribed for 6 months, and proton pump inhibitors were added for 4 weeks. All patients were followed in the outpatient clinic for 12 months after the ablation. During this follow-up period, 7-day Holter ECG recordings were performed 3, 6 and 12 months after the ablation. Additional ECGs and Holter ECG recordings were obtained when patients’ symptoms were suggestive of AF. AF recurrence was defined as a documented AF episode lasting longer than 30 seconds between 3 and 12 months after the ablation (thus, including a 3-month “blanking period”). All patients with sustained early recurring AF underwent direct cardioversion. Additional drug administration was left to the discretion of the treating physician.

**Data analysis, gene-enrichment, and pathway-enrichment**

Association of genotypes with left atrial diameter was detected using linear regression with adjustment for age, gender and AF type. Association of genotypes with AF type (persistent AF) and arrhythmia recurrence was detected using logistic regression analysis with adjustment for age and gender.

Illumina’s exome arrays contain specific “exm-SNPs” which were assigned to their corresponding dbSNP rs IDs prior further analysis.

The resulting SNP lists were used for gene enrichment. This was done with Knowledge-based mining system for Genome-wide Genetic studies (KGG)^2^. R*-*square values representing linkage disequilibrium data corresponding to the CEU (Northern Europeans from Utah) population was received from 1000 Genomes project phase 1v3 to adjust for SNP dependency. SNPs were mapped onto genes according to GenCode v23 information’s. SNPs within a range of 5kb upstream and downstream of the gene were assigned to the gene. If a SNP was in the overlapping region of two genes it was assigned to both. The KGG GATES algorithm, an extension of Simes test, was used to calculate enrichment p-values incorporating functional SNP weights controlling for LD and gene length. Enrichment p-values < 0.05 were regarded statistically significant.

For pathway enrichment analysis we used the Gene Annotation Tool to Help Explain Relationships (GATHER)^3^ and WEB-based Gene SeT AnaLysis Toolkit (WebGestalt)^4,5^ together with the databases provided by Kyoto Encyclopedia of Genes and Genomes (KEGG)^5^. Non-random over representation of genes from our candidate gene list in specific KEGG pathways was regarded significant when Fisher's exact test p-value with False Discovery Rate (GATHER) or hypergeometric distribution p-value corrected for multiple testing using Bonferroni correction (WebGestalt) was < 0.05.

We applied a two-stage analysis plan. First, we identified consistently enriched KEGG pathways in left atrial diameter and AF type present in both enrichment tools. Second, association of those identified pathway(s) with arrhythmia recurrence was tested with both enrichment tools.

**References**

1. Husser, D. *et al.* Genomic contributors to rhythm outcome of atrial fibrillation catheter ablation - pathway enrichment analysis of GWAS data. *PloS one.* **11,** e0167008 (2016).

2. Li, M.-X. Gui, H.-S. Kwan, J. S. H. & Sham, P. C. GATES: a rapid and powerful gene-based association test using extended Simes procedure. *Am. J. Hum. Genet.* **88,** 283–293 (2011).

3. Chang, J. T. & Nevins, J. R. GATHER: a systems approach to interpreting genomic signatures. *Bioinformatics (Oxford, England).* **22,** 2926–2933 (2006).

4. Zhang, B. Kirov, S. & Snoddy, J. WebGestalt: an integrated system for exploring gene sets in various biological contexts. *Nucleic Acids Res.* **33,** W741-8 (2005).

5. Kanehisa, M. Sato, Y. Kawashima, M. Furumichi, M. & Tanabe, M. KEGG as a reference resource for gene and protein annotation. *Nucleic Acids Res.* **44,** D457-62 (2016).

**Supplementary Table 1: Candidate genes associated with AF progression and recurrence involved in (A) calcium signaling pathway and (B) ECM-receptor interaction pathway.**

**(A) calcium signaling pathway**

**Gene Full name Uniprot ID**

ADCY2 adenylate cyclase 2 Q08462

ADCY3 adenylate cyclase 3 O60266

ADCY8 adenylate cyclase 8 P40145

ADRA1A adrenoceptor alpha 1A P25100/ P35348

ATP2B2 ATPase, Ca++ transporting, plasma membrane 2 P23634/ Q01814

BDKRB2 bradykinin receptor B2 P30411

CACNA1A calcium channel, voltage-dependent, P/Q type,

alpha 1A subunit O00555

CACNA1B calcium channel, voltage-dependent, N type,

alpha 1B subunit Q00975

CACNA1C calcium channel, voltage-dependent, L type,

alpha 1C subunit Q13936

CACNA1D calcium channel, voltage-dependent, L type,

alpha 1D subunit Q01668

CACNA1E calcium channel, voltage-dependent, R type,

alpha 1E subunit Q15878

CACNA1H calcium channel, voltage-dependent, T type,

alpha 1H subunit O95180

CACNA1S calcium channel, voltage-dependent, L type,

alpha 1S subunit Q13698

CALML3 calmodulin-like 3 P27482

CAMK2A calcium/calmodulin-dependent protein

kinase II alpha Q9UQM7

CAMK4 calcium/calmodulin-dependent protein kinase IV Q16566

CHRM2 cholinergic receptor, muscarinic 2 P08172

CHRM3 cholinergic receptor, muscarinic 3 P20309

CREBBP CREB binding protein Q92793

EGFR epidermal growth factor receptor P00533

ERBB4 v-erb-a erythroblastic leukemia viral

oncogene homolog 4 Q15303

GNA14 guanine nucleotide binding protein, alpha 14 O95837

GNAL guanine nucleotide binding protein, alpha

activating activity polypeptide, olfactory type P38405

GNAQ guanine nucleotide binding protein, q polypeptide P50148

GRIN1 glutamate receptor, ionotropic,

N-methyl D-aspartate 1 Q05586

GRM1 glutamate receptor, metabotropic 1 Q13255

GRM5 glutamate receptor, metabotropic 5 P41594

HRH2 histamine receptor H2 P25021

HTR2A 5-hydroxytryptamine receptor 2A,

G protein-coupled P28223

ITPR1 inositol 1,4,5-trisphosphate receptor, type 1 Q14643

ITPR2 inositol 1,4,5-trisphosphate receptor, type 2 Q14571

ITPR3 inositol 1,4,5-trisphosphate receptor, type 3 Q14573

LHCGR luteinizing hormone/choriogonadotropin receptor P22888

NFATC1 nuclear factor of activated T-cells, cytoplasmic,

calcineurin-dependent 1 O95644

NFATC2 nuclear factor of activated T-cells, cytoplasmic,

calcineurin-dependent 2 Q13469

P2RX6 purinergic receptor P2X, ligand-gated ion channel, 6 O15547

PDGFRB platelet-derived growth factor receptor,

beta polypeptide P09619

PLCB1 phospholipase C, beta 1 Q9NQ66

PLCB4 phospholipase C, beta 4 Q15147

PLCE1 phospholipase C, epsilon 1 Q9P212

PLCG2 phospholipase C, gamma 2 P16885

PRKCA protein kinase C, alpha P17252

PRKCB protein kinase C, beta P05771

PTGER3 prostaglandin E receptor 3 P43115

PTGFR prostaglandin F receptor P43088

PTK2B PTK2B protein tyrosine kinase 2 beta Q14289

PYGL phosphorylase, glycogen, liver P06737

RYR1 ryanodine receptor 1 P21817

RYR2 ryanodine receptor 2 Q92736

RYR3 ryanodine receptor 3 Q15413

SLC8A1 solute carrier family 8, member 1 P32418

SLC8A3 solute carrier family 8, member 3 P57103

SYK spleen tyrosine kinase P43405

TACR3 tachykinin receptor 3 P29371

TTN titin Q8WZ42

**B) ECM-receptor interaction pathway**

**Gene Full name Uniprot ID**

AGRN Agrin O00468

CD36 Platelet glycoprotein 4 P16671

COL4A2 Collagen alpha-2(IV) chain P08572

COL4A4 Collagen alpha-4(IV) chain P53420

COL5A1 Collagen alpha-1(V) chain P20908

COL5A3 Collagen alpha-3(V) chain P25940

DAG1 Dystroglycan Q14118

FN1 Fibronectin P02751

HSPG2 **Basement membrane-specific heparan**

**sulfate proteoglycan core protein** P98160

ITGA1 Integrin alpha-1 P56199

ITGA4 Integrin alpha-4 P13612

ITGA9 Integrin alpha-9 Q13797

ITGB3 Integrin beta-3 P05106

ITGB4 Integrin beta-4 P16144

ITGB5 Integrin beta-5 P18084

ITGB6 Integrin beta-6 P18564

LAMA1 Laminin subunit alpha-1 P25391

LAMA3 Laminin subunit alpha-3 Q16787

LAMA5 Laminin subunit alpha-5 O15230

LAMC3 Laminin subunit gamma-3 Q9Y6N6

RELN Reelin P78509

SDC2 Syndecan-2 P34741

SV2B Synaptic vesicle glycoprotein 2B Q7L1I2

TNR Tenascin-R Q92752

TNXB Tenascin-X P22105

VWF von Willebrand factor P04275
